# Utilization of Interspecific High-Density Genetic Map of RIL Population for the QTL Detection and Candidate Gene Mining for 100-Seed Weight in Soybean

**DOI:** 10.3389/fpls.2019.01001

**Published:** 2019-09-04

**Authors:** Benjamin Karikari, Shixuan Chen, Yuntao Xiao, Fangguo Chang, Yilan Zhou, Jiejie Kong, Javaid Akhter Bhat, Tuanjie Zhao

**Affiliations:** Soybean Research Institution, National Center for Soybean Improvement, Key Laboratory of Biology and Genetics and Breeding for Soybean, Ministry of Agriculture, State Key Laboratory of Crop Genetics and Germplasm Enhancement, Nanjing Agricultural University, Nanjing, China

**Keywords:** soybean, seed-weight, QTL, candidate gene, marker-assisted breeding

## Abstract

Seed-weight is one of the most important traits determining soybean yield. Hence, it is prerequisite to have detailed understanding of the genetic basis regulating seed-weight for the development of improved cultivars. In this regard, the present study used high-density interspecific linkage map of NJIR4P recombinant inbred population evaluated in four different environments to detect stable Quantitative trait loci (QTLs) as well as mine candidate genes for 100-seed weight. In total, 19 QTLs distributed on 12 chromosomes were identified in all individual environments plus combined environment, out of which seven were novel and eight are stable identified in more than one environment. However, all the novel QTLs were minor (*R*^2^ < 10%). The remaining 12 QTLs detected in this study were co-localized with the earlier reported QTLs with narrow genomic regions, and out of these only 2 QTLs were major (*R*^2^ > 10%) viz., *qSW-17-1* and *qSW-17-4*. Beneficial alleles of all identified QTLs were derived from cultivated soybean parent (Nannong493-1). Based on Protein ANalysis THrough Evolutionary Relationships, gene annotation information, and literature search, 29 genes within 5 stable QTLs were predicted to be possible candidate genes that might regulate seed-weight/size in soybean. However, it needs further validation to confirm their role in seed development. In conclusion, the present study provides better understanding of trait genetics and candidate gene information through the use high-density inter-specific bin map, and also revealed considerable scope for genetic improvement of 100-seed weight in soybean using marker-assisted breeding.

## Introduction

Soybean (*Glycine max* L. Merr.) is one of the most economically important crop being rich source of both edible oil and protein as well as has significant role in health, biofuel, and soil fertility improvement (Kulkarni et al., 2016). In China, soybean production has continuously declined with considerable low yield increase in the past 50 years (Liu et al., 2018). Moreover, China imports >80% of soybean for their total domestic use; hence, it is prerequisite to increase the domestic production of soybean to make country self-sufficient (Liu et al., 2018). Different yield-related traits are targeted by plant breeders to increase soybean production. In this context, seed-weight is one of the most important yield-related trait for increasing seed yield in soybean; however, it is a complex quantitative trait governed by polygenes and are highly influenced by environment, which makes it selection difficult for plant breeders (Yao et al., 2015). Furthermore, seed weight/size determines the specific soy-based food product that can be made from soybean (Cui et al., 2004; Gandhi, 2009). For instance, small-seeded cultivars are suitable for fermented soybean (natto) and sprout production, whereas large-seeded cultivars are used for boiled soybean (nimame), green soybean (edamame), soymilk, and soybean curd (tofu) (Liang et al., 2016; Teng et al., 2017; Wu et al., 2018). In addition, seed weight/size influences germination ability and seedling vigor, which in turn determines the competitive ability of the seedling for light, nutrient resources, and stress tolerance (Coomes and Grubb, 2003; Gomez, 2004; Haig, 2013).

Seed weight is one of the traits that was altered during domestication (Lee et al., 2011; Han et al., 2016). During domestication process from wild species to cultivated soybean, selecting desirable agronomic traits to keep achieving high yield allows many genes to be either directly selected or filtered out, resulting in a significant reduction of genetic diversity in soybean gene pool (Guo et al., 2010; Tang et al., 2010). Hyten et al. (2006) suggested that 50% of the genetic diversity and 81% of the rare alleles have been lost during domestication and that 60% of the genes show significant changes in allele frequency as a result of soybean domestication. It has been reported that wild soybean (*Glycine soja*) is an important source of genes for higher yield and related traits, quality, as well as biotic and abiotic stresses (Zhou Z. et al., 2015). Thus, it is necessary to broaden the gene pool in soybean breeding from diverse sources, especially from wild soybean (*G. soja*). The seed of cultivated soybean (*G. max*) is heavier and bigger compared to the wild accessions (Yu et al., 2017). Both wild and cultivated soybean belong to the same genus *Glycine* (Kim M.Y. et al., 2010), with the former having higher level of genetic diversity, as well as better adaptation to harsh environments (Stupar, 2010; Qiu et al., 2013; Zhang et al., 2017). Thus, *G. soja* holds great potential to improve its agriculturally important domesticated relative (*G. max*), beyond what is currently known (Kofsky et al., 2018). For example, comparative genomics, transcriptomics, and bioinformatics application have revealed the role of domestication in the seed weight of soybean (Lu et al., 2016; Zhao et al., 2016; Yu et al., 2017).

Quantitative trait loci (QTL) mapping using domesticated and wild progenitors have been reported to be the useful means for identifying genomic regions involved in morphological and physiological changes that distinguish crops from their wild relatives (Paterson, 2010). The wild soybean has been recently reported to be an important source of QTLs contributing to the increase in seed size in soybean. For example, Lu et al. (2017) identified a phosphatase 2C protein (*PP2C-1*) allele from wild soybean underlying a QTL that enhances the 100-seed weight in soybean. Although many genetic studies have been carried out in the past decades to identify QTLs for seed weight/size using different types of DNA markers through QTL mapping analyses. Currently, there are a total of 325 QTLs identified for seed-weight/size available on SoyBase^1^, and most of them are minor and not validated (Liu et al., 2018). In addition, knowledge for the molecular mechanism of soybean seed weight is very limited compared to other crops like rice (Wang et al., 2015; Liu et al., 2018). Till date, only two genes related to seed weight/size have been isolated from soybean viz., *ln* (Jeong et al., 2012) and *PP2C-1* (Lu et al., 2017). Hence, it is prerequisite to identify stable QTLs for seed-weight as well as mine candidate genes underlying them to facilitate understanding of the molecular mechanisms regulating seed-weight in soybean (Kato et al., 2014). Furthermore, only few mapping populations derived from wild and domesticated soybean crosses have been used for QTLs detection of seed-weight in soybean^1^. Also, most of the previous studies have used low-throughput markers (such as SSR) for QTL identification of seed-weight in soybean (Panthee et al., 2005; Gai et al., 2007; Kato et al., 2014; Kulkarni et al., 2016; Wu et al., 2018). These marker systems have low resolution and larger confidence interval compared with high-density SNP markers (Hyten et al., 2010; Xu et al., 2013; Lu et al., 2017) that were revealed to be useful for high-throughput QTL mapping. Also, most of the published reports did not mine the candidate genes for seed-weight (Zhang et al., 2004; Kato et al., 2014; Kulkarni et al., 2016; Wu et al., 2018).

Therefore, by keeping the above into view the present study used high-density inter-specific genetic map of the recombinant inbred line (RIL) population (NJIR4P) derived from a cross between Nannong493-1 (*G. max*) and PI 342618B (*G. soja*) that was evaluated in multiple environments to map stable QTLs as well as mine possible candidate genes underlying 100-seed weight in soybean. Using interspecific RIL population with wide range of variation in 100-seed weight has greatly assisted in the detection of more number of major and minor QTLs regulating 100-seed weight in soybean. The use of this RIL population could enhance our understanding of molecular mechanism, evolution, and genetic regulation of seed weight in soybean. The results of the present study will be helpful in marker-assisted breeding (MAB) for developing soybean varieties with improved seed-weight.

## Materials and Methods

### Plant Materials and Experimental Conditions

An interspecific RIL population consisting of 161 lines were derived through single seed descent (SSD) method by crossing a soybean cultivar Nannong493-1 (*G. max*) with wild soybean line PI 342618B (*G. soja*), and this RIL population were named as NJIR4P. The Nannong493-1 parent has a higher 100-seed weight with an average value of 18.02 ± 2.60 g, whereas PI 342618B is an annual wild soybean with low 100-seed weight (1.4 g) (Xie et al., 2014). The RILs (F_6__:__9_–F_6__:__11_) along with their parents were planted in four different environments viz., Fengyang Experimental Station, Chuzhou, Anhui Province (Latitude 32°87′ N; Longitude 117°56′ E), in 2012 (FY2012), and Jiangpu Experimental Station, Nanjing, Jiangsu Province (JP) (Latitude 33°03′ N; Longitude 118°63′ E) in 2012, 2013, and 2014 (JP2012, JP2013, and JP2014). Soybean lines were planted in a single line plot of 1 m in length and 0.5 m in width in a randomized complete block design (RCBD) with three replications. Standard cultural and agronomic practices were followed in each environment (Lihua, 1982; Liu et al., 2008).

### Phenotypic Analysis of 100-Seed Weight

Each row of the RILs and their parents were harvested, threshed, and dried to a suitable moisture. Four-hundred healthy dried seeds from each row were selected randomly for measurement of 100-seed weight. The 100-seed weight, i.e., weight of 100 seeds at 13% moisture content was measured by electronic balance and were repeated four times. Seed-weight was calculated for all the three replication and mean value was used for analysis. Analysis of variance (ANOVA) in each environment and combined environments (CEs) were conducted using the general linear model (GLM) and mixed procedure, respectively, in SAS (SAS Institute, 2010. SAS/STAT software version 9.2; SAS Institute Inc., Cary, NC, United States). The broad-sense heritability (*H*^2^) was calculated for both individual environments plus CE following the procedure of Hanson et al. (1956). Also genotypic coefficients of variation (GCV) was calculated by using the following formula proposed by Singh (1985): $\text{GCV}=\frac{\sqrt{{}^{2}\,g}}{\text{μ}}$, where $\sqrt{{\text{σ}}^{2}\,g}$ is the genotypic standard deviation in each environment while μ is the mean value of 100-seed weight.

### QTL Mapping Analysis

In the present study, an inter-specific high-density bin map earlier developed by Wang et al. (2016) by using RAD-sequencing approach for this population was used for QTL mapping. This bin map consisted of 4,354 bin markers that were derived from 80,995 single-nucleotide polymorphisms (SNPs) distributed on all 20 soybean linkage groups/chromosomes, and has a total length of 2,136.717 cM. The average number of markers per linkage group and length of linkage group was 218 and 106.84 cM, respectively, with mean distance between bins as 0.49 cM (Supplementary Table 1). Among the NJRI4P-RIL, 46.07% were inherited their genetic background from Nannong493-1, 50.06% were from PI 342618B, and the remaining 3.87% were heterozygous genotypes. The segregation ratios of each bin marker were calculated, and only few significant segregation distortion regions were identified. In NJRI4P, out of 4,354 bin markers only 1 bin showed extreme segregation distortion at *P* < 0.0001 on chromosome 2, and 2 bins exhibited segregation distortion at *P* < 0.0005 on chromosomes 7 and 19, whereas the remaining bin markers did not show significant segregation distortion (Wang et al., 2016).

The QTL analysis was performed via WinQTLCart 2.5 software (Wang et al., 2007). For the WinQTLCart 2.5 software, the model of composite interval mapping (CIM) was used with a 10 cM window at a walking speed of 1 cM. The LOD threshold was calculated using 1,000 permutations for an experimental-wise error rate of *P* = 0.05 to determine whether the QTL was significantly associated with (Churchill and Doerge, 1994). The CIM model was also used to identify the main QTLs in the CE with the same parameters as used in the individual environment. Mapping for CE was done using the Best Linear Unbiased Prediction (BLUP) values for each independent environment and across all environments by using the lme4 package in R (Bates et al., 2014). QTLs detected in different environments at the same, adjacent, or overlapping marker intervals were considered the same QTL (Palomeque et al., 2009, 2010; Qi et al., 2017). QTL naming was done following the nomenclature of McCouch et al. (1997), thus starting with “q,” followed by an abbreviation of the trait name (SW, seed weight) and the name of the chromosome, followed by the number of QTL detected on the same chromosome. The QTL genetic and physical positions based on the flanking markers with known positions were used to retrieve a number of earlier reported QTLs available on SoyBase^2^ (Williams 82.a1.v.1.1). QTLs that did not overlap with reported QTLs in both genetic and physical positions were considered as new in this study. The QTLs identified in the individual environments were presented in Venn diagram using an online tool^3^ (Oliveros, 2007).

### Candidate Gene Prediction Analysis

In this study, QTL was considered as stable when detected in at least two environments. Model genes within the genomic physical position of the stable QTLs on the soybean genome (Williams 82.a1.v.1.1) available at SoyBase^3^ were downloaded. Gene ontology (GO) enrichment analysis was conducted for all the genes within each QTL region using online GO tool^4^. Gene classification was then carried out using Web Gene Ontology (WeGO) Annotation Plotting tool, Version 2.0^5^ (Ye et al., 2018). The predicted candidate genes were further subjected to Protein ANalysis THrough Evolutionary Relationships (PANTHER) Classification System to classify proteins (and their genes) in order to facilitate high-throughput analysis according to family and subfamily, molecular function, biological process, and pathway^6^. The selected candidate genes structure analysis was carried out using http://gsds.cbi.pku.edu.cn/ (Hu et al., 2014).

## Results

### Phenotypic Variation of 100-Seed Weight

**FIGURE 1:**
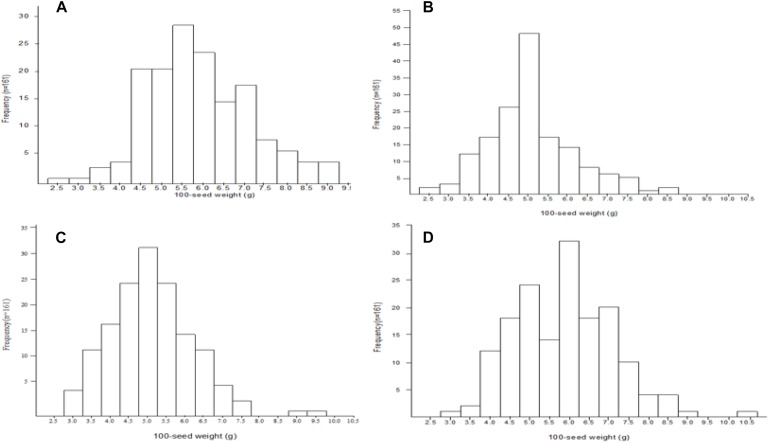
Frequency distribution of 100-seed weight QTLs among the NJIR4P-RIL in the various environments (**A**, **B**, **C**, and **D** for FY2012, JP2012, JP2013, and JP2014, respectively).

Mean, range, standard deviation, skewness, kurtosis, *H*^2^, and GCV among the RILs and their parents across the four different environments (FY2012, JP2012, JP2013, and JP2014), and CE are presented in Table 1. The average 100-seed weight of the Nannong493-1, PI 483460B, and RILs were 16.49–19.09, 1.23–1.40, and 1.37–11.84 g, respectively, across all the studied environments (Table 1). However, there was no clear transgressive segregation among the RIL (Figure 1). Furthermore, ANOVA were performed to evaluate the effects of genotypes/lines (G), environment (E), and their interactions (GE) on 100-seed weight. The RILs showed highly significant differences (*P* < 0.01) for 100-seed weight in the individual environments. ANOVA for CE showed that G, E, and GE contributed significant variation to seed weight among the RILs of NJIR4P population (Supplementary Table 2). Hence, significant influence of E and GE on 100-seed weight of soybean suggests that seed-weight is a complex quantitative trait governed by polygenes. Moreover, high *H*^2^ values in individual as well as CEs varying from 88.27 to 97.23% coupled with high GCV (>20%) suggest that considerable proportion of phenotypic variation of 100-seed weight is due to genotype.

**TABLE 1:** Descriptive statistics, broad-sense heritability (*H*^2^), and genotypic coefficient of variation (GCV) of 100-seed weight in NJIR4P RIL population and two parental lines *viz*., Nannong493-1 and PI 483460B.

**Env.**	**Parents ± SD^*a*^ (g)**		**RIL population (g)**				**Skewness**	**Kurtosis**	***H*^2^ (%)**	**GCV (%)**
						
	**Nannong493-1**	**PI483460B**	**Mean**	**Min**	**Max**	**SD**				
FY2012	19.09 ± 0.78	1.27 ± 0.06	5.64	2.12	10.23	1.35	0.52	0.24	97.23	27.54
JP2012	16.49 ± 0.78	1.23 ± 0.05	4.84	1.37	10.18	1.23	0.73	1.57	88.27	28.40
JP2013	16.88 ± 1.35	1.40 + 0.17	4.85	2.31	11.78	1.27	1.12	3.29	93.79	30.85
JP2014	17.66 ± 0.98	1.41 ± 0.05	5.64	2.53	11.84	1.44	0.54	0.92	89.13	27.37
CE^b^	17.37 ± 2.43	1.33 ± 0.09	5.25	1.37	11.84	1.39	0.71	1.10	97.16	65.24

*^*a*^Standard deviation. ^*b*^Combined environment (average from the four different environments).*

### QTL Mapping of 100-Seed Weight Using CIM

**FIGURE 2:**
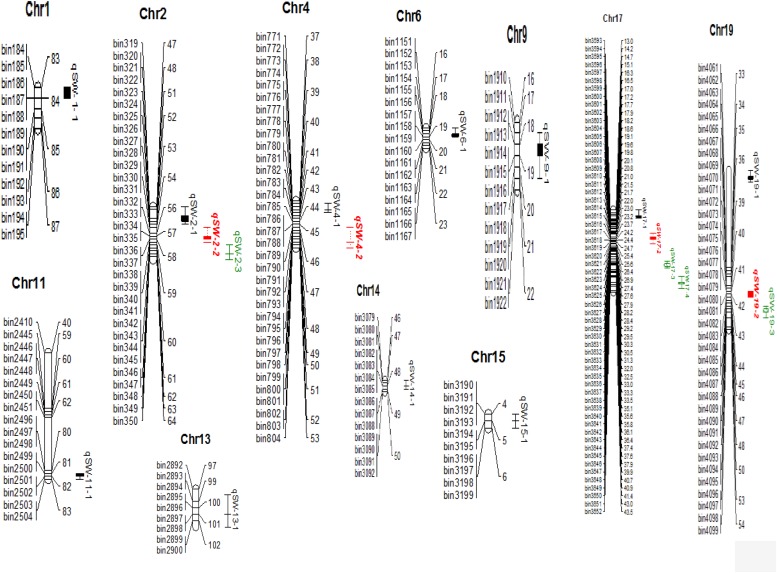
100-seed weight QTLs identified in NJIR4P-RIL (complete map is not presented here, it represents only the portion where QTLs have been identified). Right side of chromosomes indicates the interval distance between markers using cM (centiMogan) as the unit; the left side of chromosomes indicates Bin-DNA markers.

**FIGURE 3:**
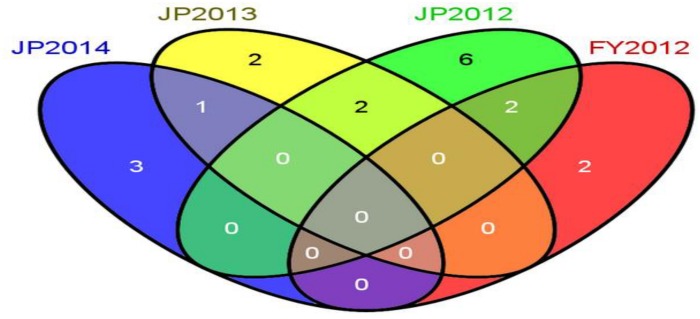
QTLs identified in different individual environments (FY2012, JP2012, JP2013, and JP2014).

A total of 19 QTLs associated with seed-weight were identified in all the individual environments plus CE distributed on 12 of the 20 chromosomes of soybean, and explaining 4.22–13.20% of the phenotypic variation (*R*^2^) (Figure 2 and Table 2). Out of these 19 QTLs, 7 were identified for the first time viz., *qSW-2-1*, *qSW-2-2*, *qSW-2-3*, *qSW-6-1*, *qSW-19-1*, *qSW-19-2*, and *qSW-19-3*, and remaining 12 QTLs have been previously reported in reference to soybean genome GmComposite2003 (SoyBase) (Table 2). The highest number of four QTLs are present on Chr17 followed by three on each Chr2 and Chr19, and the rest 10 chromosomes contain one or two QTLs each. Of the 19 QTLs identified only two are major (*R*^2^ > 10%) viz., *qSW-17-*1 and *qSW-17-*4 both are located on Chr17, and the remaining 17 QTLs identified are minor (*R*^2^ < 10%). Notably, the most prominent QTL with the highest LOD score (7.28) was identified in a 23.01 cM region on Chr17, named as *qSW-17-1*, explaining 13.20% of phenotypic variation. Five QTLs viz., *qSW-2-1*, *qSW-2-2*, *qSW-4-2*, *qSW-14-1*, and *qSW-17-4* were identified in more than one individual environments (Figure 3), and three more QTLs *viz.*, *qSW-4-1*, *qSW-17-1*, and *qSW-17-*3 were detected in one individual environment plus CE. Interestingly both major QTLs located on Chr17 (*qSW-17-*1 and *qSW-17-*4) were detected in more than one environments, suggesting the stability and consistency of these QTLs (Table 2). The remaining 11 QTLs were environment-specific QTLs identified in only one specific environment (Table 2). Out of these eight stable QTLs, two were novel QTLs identified for the first time (*qSW-2-*1 and *qSW-2-*2). All the QTLs identified for 100-seed weight in the RILs population displayed positive additive effects with positive alleles from higher seed-weight parent (Nannong493-1). Moreover, all the novel QTLs identified were minor (*R*^2^ < 10%), thus, none of the novel QTLs detected in this study was major. However, most of the previously detected QTLs were identified in a narrowed physical genomic region (Table 2). The highest number of QTLs for 100-seed weight were identified on Chr17, Chr2, and Chr19 suggest the important role of these chromosomes in governing the inheritance of seed-weight in soybean.

**TABLE 2:** Main QTLs identified in an interspecific RIL population (NJIR4P) in four different environments (FY2012, JP2012, JP2013, and JP2014) and combined environment (CE).

**QTL name^a^**	**Chr^b^**	**Pos (cM)^c^**	**LOD^d^**	***R*^2^(%)^e^**	***A*^f^**	**Phy Pos (bp)^g^**	**Flanking markers**	**CI (cM)^h^**	**Env.^i^**	**References^j^**
*qSW-1-1*	1	83.21	3.64	6.92	21.76	51,204,893–51,446,046	bin184-bin188	82.6–84.1	JP2014	*Seed weight 15-2* (Hyten et al., 2004)
*qSW-2-1*	2	50.11	3.89	7.75	22.41	9,751,130–10,665,129	bin319-bin325	47–51.5	JP2012	New
		50.11	3.85	7.71	23.16			47–52.4	JP2013	
*qSW-2-2*		57.21	4.15	8.06	23.97	11,419,866–13,045,477	bin330-bin343	54.1–59.7	JP2012	New
		57.21	3.90	7.14	24.03			56.2–59.2	FY2012	
*qSW-2-3*		62.91	3.16	5.86	25.03	13,613,400–14,313,380	bin345-bin353	60.3–65	FY2012	New
*qSW-4-1*	4	40.51	2.62	5.00	25.74	7,285,209–7,837,062	bin772-bin779	40.3–40.9	JP2012	Li et al., 2010; Zhang et al., 2015
		40.31	5.01	8.89	25.34			37.7–40.6	CE	
*qSW-4-2*		50.71	3.50	6.59	26.42	8,778,683–11,107,607	bin788-bin804	50.2–51.8	JP2012	
		50.71	2.56	4.22	27.01			50.2–52.5	FY2012	
*qSW-6-1*	6	18.01	2.70	4.60	27.02	3,772,133–4,753,088	bin1153-bin1159	16–19.1	JP2012	New
*qSW-9-1*	9	17.91	2.53	4.72	28.66	3,096,080–4,376,263	bin1910-bin1920	16.7–21.1	JP2012	*Seed weight 35-6* (Han et al., 2012)
*qSW-11-1*	11	81.41	4.72	8.57	30.51	35,912,819–36,898,349	bin2498-bin2503	80.6–82.7	FY2012	*Seed weight 37-9* (Teng et al., 2009; Sun et al., 2012)
*qSW-13-1*	13	100.81	2.82	5.35	30.53	32,932,681–33,843,851	bin2893-bin2900	98.3–102.4	JP2013	*Seed weight 45-6* (Yan et al., 2014)
*qSW-14-1*	14	47.61	4.14	7.87	31.04	9,463,148–13,115,201	bin3080-bin3087	46.6–48.8	JP20120	*Seed weight 36-14* (Han et al., 2012)
		47.91	3.71	7.08	31.11			46.3–48.7	JP2013	
*qSW-15-1*	15	4.51	2.81	4.91	25.74	1,499,442–2,546,339	bin3190-bin3199	3.7–6.2	JP2013	
*qSW-17-1*	17	23.21	5.65	10.87	32.33	5,762,020–6,841,677	bin3613-bin3621	22.8–25.6	JP2012	
		23.01	7.28	13.20	32.12			21.8–24.8	CE	
*qSW-17-2*		29.51	3.75	7.51	32.86	7,615,994–7,984,614	bin3629-bin3631	29.2–30.3	JP2014	
*qSW-17-3*		33.51	3.05	6.09	34.15	8,235,618–8,924,784	bin3634-bin3639	32–34.5	JP2012	
		33.01	4.23	8.00	33.92			32.2-33.1	CE	
*qSW-17-4*		39.91	3.70	7.09	36.63	9,420,885–10,095,969	bin3645-bin3651	37.7–42.3	JP2013	
		40.71	5.96	11.58	37.03			39.7–42.2	JP2014	
*qSW-19-1*	19	1.91	2.86	4.87	37.91	1–906,420	bin3996-bin3999	0–4	JP2012	New
*qSW-19-2*		41.41	4.60	8.78	38.11	37,530,933–38,131,800	bin4078-bin4083	41.1–43	JP2014	New
*qSW-19-3*		46.91	3.06	5.98	43.09	38,456,684–39,675,684	bin4088-bin4095	46–50.3	JP2014	New

*^*a*^QTLs detected in different environments at the same, adjacent, or overlapping marker intervals were considered the same QTL. ^*b*^Position of the QTL. ^*c*^Chromosome on which QTL was detected. ^*d*^The log of odds (LOD) value at the peak likelihood of the QTL. ^*e*^Phenotypic variance (%) explained by the QTL. ^*f*^Additive shows beneficial alleles from parent Nannong 493-1. ^*g*^The physical position of QTL relation to soybean cultivar W82.a1.v.1.1. ^*h*^1-LOD support confidence intervals (confidence interval length). ^*i*^Environment where CE represents combined environments using the BLUP values and others refer materials and methods. ^*j*^Overlapping references.*

### Gene Ontology and Candidate Gene Prediction Within Stable QTLs

**FIGURE 4:**
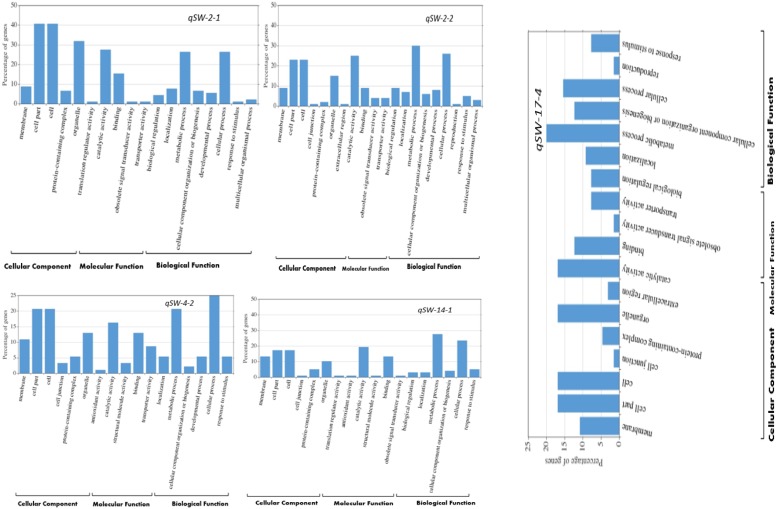
WeGO analysis of the genes located within the stable QTL regions viz., *qSW-2-1*, *qSW-2-2*, *qSW-4-2*, *qSW-14-1*, and *qSW-17-4*.

Based on the number of individual environments QTL were detected, we selected five stable QTLs identified in more than one individual environments viz., *qSW-2-1*, qSW*-2-2*, *qSW-4-2*, *qSW-14-1*, and qSW*-17-4* for GO and candidate gene prediction analysis. Within the physical genomic interval of *qSW-2-1*, qSW*-2-2*, *qSW-4-2*, *qSW-14-1*, and qSW*-17-4*, the 91, 100, 92, 137, and 70 model genes were present, respectively, and these genes as well as their gene annotation were downloaded from Soybase^7^. After GO enrichment analysis, we employed WeGO web-based tool to visualize the biological process, molecular function, and cellular component main categories (Figure 4). In all the five stable QTLs viz., *qSW-2-1*, qSW*-2-2*, *qSW-4-2*, *qSW-14-1*, and qSW*-17-4*, higher percentage of genes were associated with the terms cell part, cell, organelle, catalytic activity, binding, metabolic process, and cellular process (Figure 4). This suggests an important role of these terms in the seed development of soybean.

However, to identify the possible candidate genes underlying the above five stable QTLs responsible for 100-seed weight in soybean, we used PANTHER analysis, gene annotation information, and literature search. The PANTHER analysis is a comprehensive system that combines gene function, ontology, pathways, and statistical analysis tools, and enable biologists to analyze large-scale, genome-wide data from sequencing, proteomics, or gene expression experiments (Huaiyu et al., 2013). Based on the PANTHER analysis, gene annotation, as well as available literature, 29 genes out of total 490 model genes within the physical regions of the five stable QTLs were considered as possible candidate genes regulating seed-weight in soybean. Out of these 29 genes, 5 belong to ubiquitin-protein ligase (PC00234) class, 4 to carbohydrate transporter (PC00067), 3 are transporters (PC00227), 2 are involved in vesicle coat protein (PC00235), 1 in the SNARE protein (PC00034), and the remaining 18 belong to one or two other protein class (Table 3). Furthermore, *Glyma02g13350*, *Glyma14g12220*, and *Glyma17g13000* genes had no protein class according to PANTHER analysis, and therefore were further analyzed using the gene expression data (RNA-seq) from phytozome database^8^, and their expression data revealed that these genes were highly expressed in the seed, and thus were also included as potential candidate genes. For instance, *Glyma14g12220* has the domain of *PP2C* which is homolog to the *PP2C* that was demonstrated to enhance 100-seed weight by Lu et al. (2017).

**TABLE 3:** Thirty-one possible candidate genes predicated within five stable QTL regions identified in this study based on PANTHER analysis, gene annotation, and available literature.

**QTL**	**Mapped IDs**	**PANTHER family/subfamily**	**PANTHER protein class**
*qSW-2-1*	Glyma02g11570	E3 UBIQUITIN-PROTEIN LIGASE MARCH6 (PTHR13145:SF0)	Ubiquitin-protein ligase (PC00234)
	Glyma02g11850	CULLIN-3A-RELATED (PTHR11932:SF95)	Ubiquitin-protein ligase (PC00234)
	Glyma02g11960	RING/FYVE/PHD ZINC FINGER SUPERFAMILY PROTEIN (PTHR23012:SF165)	Ubiquitin-protein ligase (PC00234)
	Glyma02g12030	BETA-1,3-GALACTOSYLTRANSFERASE GALT1 (PTHR11214:SF129)	Glycosyltransferase (PC00111)
	Glyma02g12351	EMP24/GP25L/P24 FAMILY/GOLD FAMILY PROTEIN-RELATED (PTHR22811:SF47)	Transfer/carrier protein (PC00219); vesicle coat protein (PC00235)
	Glyma02g12390	VESICLE TRANSPORT V-SNARE 12 (PTHR21230:SF57)	SNARE protein (PC00034)
*qSW-2-2*	Glyma02g13210	SUBFAMILY NOT NAMED (PTHR24298:SF315)	Oxygenase (PC00177)
	Glyma02g13350	PROTEIN CRABS CLAW (PTHR31675:SF1)	Plant-specific transcription factor YABBY family protein^∗^
	Glyma02g13401	AGAMOUS-LIKE MADS-BOX PROTEIN AGL3 (PTHR11945:SF352)	MADS box transcription factor (PC00250)
	Glyma02g13420	AGAMOUS-LIKE 79-RELATED (PTHR11945:SF244)	MADS box transcription factor (PC00250)
	Glyma02g13730	SUBFAMILY NOT NAMED (PTHR23500:SF144)	Carbohydrate transporter (PC00067)
*qSW-4-2*	Glyma04g10590	SUBFAMILY NOT NAMED (PTHR11206:SF194)	Transporter (PC00227)
	Glyma04g10600	SUBFAMILY NOT NAMED (PTHR22951:SF24)	Vesicle coat protein (PC00235)
	Glyma04g10610	E3 UBIQUITIN-PROTEIN LIGASE ATL15-RELATED (PTHR14155:SF296)	Ubiquitin-protein ligase (PC00234)
	Glyma04g11060	PROTEIN DETOXIFICATION 10-RELATED (PTHR11206:SF87)	Transporter (PC00227)
	Glyma04g11080	MITOCHONDRIAL ADENINE NUCLEOTIDE TRANSPORTER BTL2-RELATED (PTHR24089:SF292)	Amino acid transporter (PC00046); calmodulin (PC00061); mitochondrial carrier protein (PC00158); transfer/carrier protein (PC00219)
	Glyma04g11120	SUGAR TRANSPORT PROTEIN 5 (PTHR23500:SF44)	Carbohydrate transporter (PC00067)
	Glyma04g11130	SUGAR TRANSPORT PROTEIN 5 (PTHR23500:SF44)	Carbohydrate transporter (PC00067)
	Glyma04g11140	SUGAR TRANSPORT PROTEIN 5 (PTHR23500:SF44)	Carbohydrate transporter (PC00067)
	Glyma04g12120	MITOCHONDRIAL IMPORT INNER MEMBRANE TRANSLOCASE SUBUNIT TIM44 (PTHR10721:SF1)	Mitochondrial carrier protein (PC00158); transfer/carrier protein (PC00219)
*qSW-14-1*	Glyma14g11780	TRANSMEMBRANE 9 SUPERFAMILY MEMBER 10-RELATED (PTHR10766:SF46)	Transporter (PC00227)
	Glyma14g12110	SUBFAMILY NOT NAMED (PTHR23051:SF2)	Transfer/carrier protein (PC00219)
	Glyma14g12120	MONOGALACTOSYLDIACYLGLYCEROL SYNTHASE 1, CHLOROPLASTIC (PTHR43025:SF3)	Acetyltransferase (PC00038); glycosyltransferase (PC00111); transfer/carrier protein (PC00219)
	Glyma14g12405	SEC31 ORTHOLOG, ISOFORM D (PTHR13923:SF11)	Vesicle coat protein (PC00235)
	Glyma14g12220		Phosphatase 2C (PP2C) protein^∗^
	Glyma14g13011	RING/FYVE/PHD ZINC FINGER SUPERFAMILY PROTEIN (PTHR23012:SF165)	Ubiquitin-protein ligase (PC00234)
*qSW-17-4*	Glyma17g12910	ABC TRANSPORTER G FAMILY MEMBER 32 (PTHR19241:SF280)	ATP-binding cassette (ABC) transporter (PC00003)
	Glyma17g13000	HISTONE DEACETYLASE 15 (PTHR45634:SF12)	^∗^
	Glyma17g13050	DNA MISMATCH REPAIR PROTEIN MSH2 (PTHR11361:SF35)	DNA-binding protein (PC00009)

*Protein with ^∗^ indicates these proteins are selected from literature only.*

## Discussion

### Phenotypic Analysis of Seed-Weight

Seed-weight is an important economical trait controlling the yield in soybean. Therefore, developing soybean cultivars with improved seed-weight was the prime objective of soybean breeders. However, to develop the soybean cultivars with improved seed-weight, it is necessary to understand the genetic mechanisms as well as identify genetic elements associated with 100-seed weight. Seed-weight is a polygenic quantitative trait governed by multiple genes, and is highly environmentally sensitive trait. Although over the past decades many QTLs related to soybean seed-weight/size have been reported, and there are ∼325 QTLs documented for seed weight/size in the USDA Soybean Genome Database (SoyBase^9^). However, most of these QTLs were not stable as well as confirmed due to small sized mapping population and low-density genetic map, and hence have not been used for breeding improved seed-weight in soybean. Therefore, the aim of the present study was to utilize interspecific high-density linkage map of NJRI4P RIL population evaluated in four different environments to identify the stable QTLs as well as mine possible candidate genes for 100-seed weight in soybean. In the present study, ANOVA revealed that 100-seed weight was significantly affected by G, E, and G × E, similar as reported earlier by Fasoula et al. (2004). The RIL did not show clear transgressive segregation in any of environment, that might be due to unwanted linkages between beneficial and undesirable alleles contributed by exotic germplasm (Concibido et al., 2003; Wang et al., 2014). Furthermore, the cultivated and wild parents of RIL population showed clear and large difference in seed-weight/size confirming earlier reports that 100-seed is a domestication-related trait (Zhou L. et al., 2015; Zhou Z. et al., 2015). This wide difference between two parents of inter-specific RIL population for 100-seed weight has allowed detection of more number of QTLs including some novel QTLs. Maximum 100-seed weight of the RILs in each environment was more than three times higher than that of wild parent (PI 483460B), and also the RILs with minimum seed-weight were higher than PI 483460B indicating the usefulness of wild soybean in breeding program for specific seed size (Concibido et al., 2003; Hyten et al., 2006; Kim M.Y. et al., 2010; Lam et al., 2010; Yu et al., 2017; Kofsky et al., 2018). The higher *H*^2^-value observed for seed-weight in both the individual and CEs suggests that large proportion of trait variation is under genetic control, and these findings are similar as reported earlier by Kulkarni et al. (2016).

### Genetic Control of Seed-Weight

As discussed above, many QTLs have been reported for seed-weight in soybean^10^. But majority of these previous studies used low-density genetic maps based on SSR or other low-throughput markers (Specht et al., 2001; Hyten et al., 2004; Panthee et al., 2005; Liu et al., 2011; Yao et al., 2015), which has low resolution with large confidence interval of QTLs not suitable for candidate gene detection (Hyten et al., 2010; Xu et al., 2013). The quality of genetic maps has great influence on the accuracy of QTL detection (Gutierrez-Gonzalez et al., 2011). In this context, high-density genetic map could identify more recombination events in a population, and will increase accuracy of QTL mapping (Xie et al., 2010). In the present study, we used high-density inter-specific bin map of NJIR4P RIL population consisting of 4,354 bin markers distributed to all 20 chromosomes of soybean with an average number of markers and distance per chromosome are 218 and 106.84 cM, respectively. The average distance between two markers was 0.49 cM (Wang et al., 2016). In addition, high-density genetic map assists in identifying tightly linked markers associated with QTLs, and provided a good foundation for analyzing quantitative traits. Moreover, the use of interspecific population would also enhance identification of genomic region(s) which was/were altered during domestication (Liu et al., 2018).

The QTLs associated with seed-weight in soybean has been mapped on all soybean linkage groups/chromosomes. In the present study, we identified a total of 19 QTLs associated with 100-seed weight using inter-specific genetic map of NJIR4P population, and these QTLs contributed significantly to the seed-weight. By comparing our QTL results with SoyBase database^11^, 12 QTLs have been previously reported in the same physical genomic region, and only 7 were novel identified for the first time (Table 2). The seven novel QTLs detected indicating the distinct genetic architecture of NJIR4P population, and suggest the need to use more germplasm for revealing the complex genetic basis of 100-seed weight in soybean. The physical interval of *qSW-2-1*, *qSW-2-2*, *qSW-2-3*, *qSW-6-1*, *qSW-19-1*, *qSW-19-2*, and *qSW-19-3* did not overlap with any of the previously reported seed-weight QTLs, and hence were considered as novel QTLs. The *qSW-1-1* was identified in the genetic interval (82.6–84.1 cM) that overlap with the seed-weight QTLs viz., *Seed weight 15-2* and *Seed weight 18.1-2* identified in the same genetic and physical position as reported earlier (Hyten et al., 2004; Panthee et al., 2005). Similarly, two QTLs identified on Chr4 viz., *qSW-4-1* and *qSW-4-2* overlapped with seed weight 47-1 corresponding to physical position of 96,434–51,252,852 bp (Li et al., 2010) and seed weight per plant 6-2 corresponding to physical position of 486,057–526,777 bp (Yao et al., 2015), respectively. The *qSW-9-1* were detected in the same genomic physical interval as previously reported QTL, *Seed weight 35-6* QTL (Han et al., 2012). Likewise, *SW-11-1* was located in the genomic position of *Seed weight 10-3* (Specht et al., 2001), *Seed weight 32-1* (Li et al., 2008), and *Seed weight 36-11* (Han et al., 2012). The *qSW-14-1* could be the same QTL as *Seed weight 36-14* (Han et al., 2012). Lu et al. (2017) identified QTL on Chr15 at the same physical interval (1,901,425–2,855,666 bp) as *qSW-15-*1 reported in the present study. The major and stable QTL viz., *qSW-17-1* overlapped with earlier reported QTLs, *Seed weight 21-1* (Gai et al., 2007), *Seed weight 22-3* (Zhang et al., 2004), and *Seed weight 47-2* (Li et al., 2010). Moreover, *qSW-17-2* and *qSW-17-3* overlapped with seed-weight QTLs previously reported by Li et al. (2010) and Wang et al. (2015), respectively. Another major and stable QTL identified on Chr17 viz., *qSW-17-4* has been also reported by the number of earlier studies (Kim H.-K. et al., 2010; Kato et al., 2014; Wang et al., 2015; Zhou Z. et al., 2015; Liu et al., 2018). The seven novel QTLs identified for 100-seed weight together explained ∼46% of the phenotypic variation, which suggested potential importance of these loci for seed-weight. The QTLs identified in this study had narrow genetic and physical regions for the instance, *qSW-17-4* which overlapped with *Seed weight 47-2* (Li et al., 2010). In our study, *qSW-17-4* was detected at genetic and physical positions of 37.7–42.3 cM and 9,420,885–10,095,969 bp, respectively, compared to *Seed weight 47-2* (24.52–124.30 cM and 5,788,551–40,525,673 bp). In plant breeding, stability of QTL is essential for their use in MAB. Besides, two novel stable QTLs (*qSW-2-1* and *qSW-2-2*) identified in the present study, the 12 QTLs for 100-seed weight have been previously co-localized in the same physical interval by earlier studies (see references in Table 2). Of the 12 QTLs previously reported, two are major QTLs with *R*^2^-value > 10% both located on Chr17 viz., *qSW-17-1* and *qSW-17-4* (see references in Table 2). Hence, these QTLs might also be considered as stable QTLs, and major stable QTLs can be used for further fine mapping and map-based cloning to unravel the mechanisms of seed-weight in soybean, as well as might be good for MAB. All the beneficial/positive alleles in the NJIR4P RIL population were derived from the cultivated soybean (Nannong 493-1), indicating that seed-weight was altered during domestication (Zhou L. et al., 2015; Zhou Z. et al., 2015; Lu et al., 2016). Similar to our findings, Lu et al. (2017) also reported that all the beneficial alleles for 100-seed weight were inherited from the cultivated soybean except one beneficial QTL allele viz., *PP2C-1* that was derived from wild soybean parent. Although it has been revealed that wild soybean is a potential source for improving cultivated soybean in terms of yield-related traits, seed quality, and biotic and abiotic stress tolerance (Tuyen et al., 2010; Kim et al., 2011). In accordance with the earlier studies (Wang et al., 2016; Xin et al., 2016; Liu et al., 2018), our study also revealed that alleles derived from wild soybean contribute to a reduction in seed weight in all 19 seed-weight QTLs. It is not always the purpose of soybean breeders to increase seed weight/size, but also sometimes breeding program requires a suitable/optimized combination of yield-related parameters such as seed size, the number of seeds per pod, and the number of pods per plant. Hence, QTLs detected in our study would be valuable for controlling seed size via genomic breeding by design and positional cloning of the relevant genes. Furthermore, most of the QTLs detected in this study overlapped earlier reported QTLs indicating the accuracy of our mapping results. Moreover, those confirmed in this study with narrow regions could be integrated into breeding program via marker-assisted selection (MAS).

### Candidate Gene Analysis for Seed-Weight

It is of great interest for both theoretical study and practical breeding program to identify the actual candidate gene underlying the QTL region. Most of the earlier QTL mapping on seed-weight did not mine for candidate genes (Zhang et al., 2004; Kato et al., 2014; Wu et al., 2018), and till date only two seed weight/size-related genes have been isolated from soybean viz., *ln* gene has a large effect on the number of seeds per pod and seed size (Jeong et al., 2012), and recently, the *PP2C-1* (protein phosphatase type-2C) allele from wild soybean accession ZYD7 was found to contribute to the increase in seed size (Lu et al., 2017). Hence, based on the available information in current literature, gene annotation as well as bioinformatics tools, the present study identified the possible candidate genes regulating the 100-seed weight in soybean that underlie the stable QTLs. A total of 490 model genes were mined from the physical regions of the five stable QTLs viz., *qSW-2-1*, *qSW-2-2*, *qSW-4-2*, *qSW-14-1*, and *qSW-17-4*, and out of these 29 were considered as possible candidate genes based on the PANTHER analysis, gene function, and available literature (Huaiyu et al., 2013). Based on the WeGo analysis most of the genes underlying above five stable QTLs belong to the terms cell component, catalytic activity, binding, transporting, metabolic and cellular process, and these elements were reported to be vital in seed development (Fan et al., 2006; Mao et al., 2010; Li and Li, 2014). For example, *Glyma02g13210* gene underlying QTL *qSW-2-2* belongs to oxygenase (PC00177) protein class, that has been demonstrated to regulate seed size in soybean (Zhao et al., 2016). Similarly, protein family E3 ubiquitin-protein ligase (PC00234) are involved in the ubiquitin-proteasome pathway, and this protein family include members from various crop species such as *DA1*, *DAR1*, *DA2*, and *EOD1/BB* (Arabidopsis), *GW2* (rice), *TaGW2* (Wheat), *ZmGW2* (maize), and *UBP15/SOD2* (Arabidopsis), and all these genes have been reported to have significant effect on seed development (Li and Li, 2014, 2016; Ge et al., 2016). Thus, *Glyma02g11570*, *Glyma02g11850*, *Glyma02g11960*, *Glyma04g10610*, and *Glyma14g13011* belonging to E3 ubiquitin-protein ligase (PC00234) were considered as possible candidate genes in the present study. Furthermore, Xian-Jun et al. (2007) reported a gene underlying QTL for rice grain width and weight (*GW2*) that encodes a previously unknown RING-type E3 ubiquitin ligase has been demonstrated by Xian-Jun et al. (2007). They demonstrated that loss of *GW2* function increased cell numbers, resulting larger spikelet hull, and accelerated the grain milk filling rate, resulting in enhanced grain width, grain weight, and yield. It has been revealed that regulation of seed development is controlled by source (leaf) and sink (seed) relationship in plants (Schnyder, 1993), which is influenced by assimilate translocation/transportation. Therefore, genes viz., *Glyma02g13730*, *Glyma04g10590*, *Glyma04g11060*, *Glyma04g11120*, *Glyma04g11130*, *Glyma04g11140*, and *Glyma14g11780* belonging to carbohydrate transporter (PC00067 or PC00227) gene family were might be possible candidate genes for seed-weight. Legume seed development is closely related to metabolism and nutrient (sucrose) transport (Borisjuk et al., 2003). Candidate genes *Glyma02g12351*, *Glyma04g10590*, *Glyma04g10600*, and *Glyma14g12405* belong to vesicle coat protein (PC00235). This protein family have been reported to be involved in protein–protein interaction and transport (Harley and Beevers, 1989; Anantharaman and Aravind, 2002). Two candidate genes *Glyma02g13401* and *Glyma02g13420* were members of K and MAD box protein family, and this protein family has been reported to regulate flower development in plants (Bowman et al., 1991; Ditta et al., 2004). The flower as an organ acts as either source or sink and determine the seed number, which indirectly affect the seed size and shape (Stanton, 1984; Jia et al., 2016). The *Glyma04g11080* belongs to several protein classes such as amino acid transporter (PC00046); calmodulin (PC00061); mitochondrial carrier protein (PC00158); transfer/carrier protein (PC00219) which could possibly be involved in seed weight regulation. For example, in rice, Asano et al. (2002) has shown that a calmodulin-like domain protein kinase is required for storage product accumulation during seed development. Moreover, *Glyma02g12030*, *Glyma04g12120* and *Glyma14g12120* belong to one or more protein classes: such as acyltransferase (PC00038), glycosyltransferase (PC00111), and transfer/carrier protein (PC00219), and these protein classes were demonstrated to play role in seed development (Rehman et al., 2016). The *Glyma17g12910* gene underlying a major stable QTL, *qSW-17-4*, belongs to ATP-binding cassette (ABC) transporter (PC00003) which could possibly be involved in seed development (David et al., 2010). In addition, *Glyma17g13000* belongs to histone deacetylase 15 (PTHR45634:SF12) that might be involved in regulating seed weight (Shahbazian and Grunstein, 2007; Peserico and Simone, 2011). As Yang et al. (2016) demonstrated maize histone deacetylase HDA101 function and regulatory mechanism during seed development. Also, *Glyma17g13050* and Glyma17g13210 which code for DNA-binding protein (PC00009) and leucine-rich repeat-containing protein, respectively, play significant role in seed development (Li and Li, 2016; Li et al., 2019). Among the predicted candidate genes, the minimum number of exons and introns was two with the maximum gene sequence of 13,670 bps for *Glyma17g13050* (Supplementary Figure 1). However, few of the 29 possible candidate genes predicted in this study for 100-seed weight have been included in our on-going projects for their functional validation. Lastly, the major and stable QTLs identified in the present study will be the main focus of soybean breeders for fine mapping and MAB of soybean cultivars with improved 100-seed weight.

## Conclusion

In conclusion, the present study used high-density bin map of an interspecific RIL population (NJIR4P) evaluated in multiple environments to detect QTLs as well as mine possible candidate genes controlling 100-seed weight. A total of 19 QTLs were found associated with 100-seed weight, and out of which 7 were novel (reported for the first time). In addition, out of 19 QTLs, 8 were considered as stable QTLs identified in either more one individual environments or one individual environment plus CE, and two of them were major viz., *qSW-17-1* and *qSW-17-4* (*R*^2^ > 10%). Moreover, most of the previously reported QTLs validated in the present study had narrow physical genomic interval. All the beneficial/positive alleles of 19 QTLs were derived from the cultivated soybean (Nannong493-1). Twenty-nine possible candidate genes were mined within the five stable QTLs and most of them are belonging to ubiquitin-protein ligase (PC00234) that have been earlier reported to play significant role in seed/organ size development and regulation. However, it needs further validation to determine their actual role in seed weight and development, although few of them have been included in our on-going projects for functional validation. Hence, after proper functional validation of these candidate genes, these candidate genes can be used for improving 100-seed weight of soybean through transgenic or MAB. Lastly, our study provides detailed information for accurate QTL localization and candidate gene discovery, and these findings will be of great use for MAS of soybean varieties with improved seed-weight.

## Data Availability

All datasets generated for this study are included in the manuscript and/or the Supplementary Files.

## Author Contributions

TZ conceived and designed the experiments. BK, SC, YX, FC, YZ, and JK performed the experiments. BK and JB analyzed the data. BK and JB drafted the manuscript. TZ and JB revised the manuscript.

## Conflict of Interest Statement

The authors declare that the research was conducted in the absence of any commercial or financial relationships that could be construed as a potential conflict of interest.

## Footnotes

1 http://www.soybase.org/

2 www.soybase.org

3 http://bioinfogp.cnb.csic.es/tools/venny_old/venny.php

4 http://geneontology.org/

5 http://wego.genomics.org.cn/

6 http://pantherdb.org/

7 www.soybase.org

8 https://phytozome.jgi.doe.gov/

9 http://www.soybase.org

10 www.soybase.org

11 www.soybase.org
