# Supplementary material for: Utilization of Interspecific High-Density Genetic Map of RIL Population for the QTL Detection and Candidate Gene Mining for 100-Seed Weight in Soybean
Source: Front Plant Sci. 2019 Sep 4;10:1001. doi: 10.3389/fpls.2019.01001 (PMC6737081; doi:10.3389/fpls.2019.01001)
Supplement: Supplementary file 1 [file Table_1.DOCX]

**Supplementary Materials**

**Supplementary Figure 1:** Gene structure analysis of possible **c**andidate genes identified in the present study.

**Supplementary Table 1:** Detail information on the high-density interspecific bin map of NJIR4P RIL population used for this study.

**Supplementary Table 2:** Analysis of variance (ANOVA) for the combined environment (CE).

**Supplementary Figure 1**


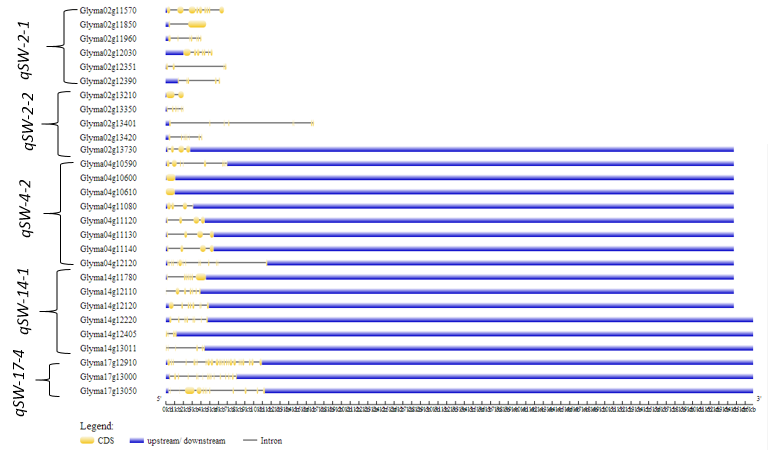


### Glyma04g11060 has no corresponding sequence data on <https://phytozome.jgi.doe.gov> (*Glycine max Wm82.a2.v1* (Soybean)

**Supplementary Table 1:** Detail information on the high-density interspecific bin map of NJIR4P RIL population used for this study.

| **Chro** | **Distance (cM)** | **No. of Markers** |
| --- | --- | --- |
| 1 | 101.00 | 225 |
| 2 | 127.37 | 258 |
| 3 | 99.94 | 216 |
| 4 | 105.83 | 225 |
| 5 | 101.09 | 195 |
| 6 | 123.70 | 238 |
| 7 | 105.03 | 226 |
| 8 | 135.47 | 294 |
| 9 | 101.01 | 214 |
| 10 | 125.35 | 237 |
| 11 | 88.28 | 191 |
| 12 | 97.44 | 208 |
| 13 | 144.77 | 259 |
| 14 | 87.95 | 194 |
| 15 | 104.69 | 209 |
| 16 | 85.65 | 172 |
| 17 | 103.13 | 220 |
| 18 | 100.81 | 212 |
| 19 | 99.38 | 201 |
| 20 | 98.82 | 160 |
| **Total** | **2136.72** | **4354** |
| Average markers per chromosome | | 218 |
| Average distance between two bin markers | | 0.49 |
| Average distance per chromosome | | 217.70 |

### Supplementary Table 2: Analysis of variance (ANOVA) for the combined environment (CE).

| **Sources of variation** | **DF** | **SS** | **MS** | ***F-*value** | **Pr>F** |
| --- | --- | --- | --- | --- | --- |
| Block within | 8 | 45.13 | 5.64 | 10.82 | < 0.0001 |
| Genotype | 160 | 1876.72 | 11.73 | 22.49 | < 0.0001 |
| Environment | 3 | 296.28 | 98.76 | 189.36 | < 0.0001 |
| Genotype×Environment | 480 | 575.68 | 1.2 | 2.3 | < 0.0001 |
| Error | 1140 | 594.57 | 0.53 |  |  |
